# Immunizations in children exposed perinatally to HIV – from theory to practice

**DOI:** 10.1186/1471-2334-14-S4-P2

**Published:** 2014-05-29

**Authors:** Delia Adelina Vlad, Mariana Mărdărescu, Cristina Petre, Ruxandra Neagu-Drăghicenoiu, Rodica Ungurianu, Sorin Petrea, Ana Maria Tudor, Alina Cibea

**Affiliations:** 1 National Institute for Infectious Diseases “Prof. Dr. Matei Balş”, Bucharest, Romania

## 

Increased vulnerability of HIV infected children to numerous infections argues vaccination to protect them. Lately we faced diseases preventable by vaccination in children perinatally exposed to HIV infection and, prematurity, abandonment, social condition, lack of understanding, drug mothers are among the causes that prevent children from immunization HIV-positive women.

During 01.01.2011-31.12.2013, in the department of immunocompromised children at the National Institute for Infectious Diseases “Prof. Dr. Matei Balş” we conducted an analysis of immunizations to 198 children exposed perinatally to HIV infection aged 0-18 months. Data from medical records and history refers to the share of vaccinations starting maternity and continuing thereafter through family physicians or pediatricians within hospital units caring for these children. A major role is parents’ avoidance to health services. Most of them rely on various reasons to avoid contact with health workers, so that most children do not benefit from prevention through vaccination.

A percentage of 79.29% were vaccinated in hospital. Only 63.13% were BCG vaccinated, prematurity and knowing immunological status (CD4) representing key factors for deprivation of BCG vaccination. Optional vaccinations are hard supported by parents, such as 5.94% children were vaccinated for flu, RSV, 3.96%, 1.98% and Prevenar Rotarix. We found an increased incidence of RSV, rotavirus enterocolitis, pneumococcal pneumonia and otitis, and measles, often evolving severely.

HIV perinatally exposed infants need protection against vaccine-preventable diseases. Immunization does not influence disease progression, but the lack of vaccination can lead to severe infection, potentially fatal.

